# Improving cellulosic ethanol production by an engineered yeast consortium displaying a pentafunctional mini-cellulosome

**DOI:** 10.1093/femsyr/foaf022

**Published:** 2025-05-21

**Authors:** Xiaofei Song, Jianze Zhang, Siyu Fu, Ziyi Liu, Yan Chen, Tingheng Zhu

**Affiliations:** College of Biotechnology and Bioengineering, Zhejiang University of Technology, Hangzhou 310014, Zhejiang Province, China; College of Biotechnology and Bioengineering, Zhejiang University of Technology, Hangzhou 310014, Zhejiang Province, China; College of Biotechnology and Bioengineering, Zhejiang University of Technology, Hangzhou 310014, Zhejiang Province, China; College of Biotechnology and Bioengineering, Zhejiang University of Technology, Hangzhou 310014, Zhejiang Province, China; College of Biotechnology and Bioengineering, Zhejiang University of Technology, Hangzhou 310014, Zhejiang Province, China; College of Biotechnology and Bioengineering, Zhejiang University of Technology, Hangzhou 310014, Zhejiang Province, China

**Keywords:** mini-cellulosome, ethanol, cellulose, *Saccharomyces cerevisiae*, consortium

## Abstract

As a traditional ethanol-producing microorganism, *Saccharomyces cerevisiae* is an ideal host for consolidated bioprocessing. However, when overloaded cellulase genes are expressed in yeast, the metabolic burden on cells may greatly affect cell growth and cellulosic ethanol production. In this study, we developed a yeast consortium system that secretes and assembles five types of cellulases on the yeast cell surface to improve cellulosic ethanol production. This system involves one display strain, which provides the scaffoldin on the surface and several secretion strains that secrete each cellulase. The secreted dockerin-containing enzymes, cellobiohydrolase (CBH), endoglucanase (EG), β-glucosidase (BGL), cellobiose dehydrogenase (CDH), and lytic polysaccharide monooxygenase (LPMO), were randomly assembled to the scaffoldin to generate a pentafunctional mini-cellulosome via cohesion–dockerin interactions. The developed system relieved the metabolic burden placed on the engineered single yeast strain and leveraged the innate metabolic potential of each host. In addition, the enzymes in the consortium acted synergistically and efficiently boosted cellulose degradation and ethanol production. When compared with the conventional system, this consortium system increased the ethanol titers from 2.66 to 4.11 g/l with phosphoric acid swollen cellulose (PASC) as the substrate, an improvement of 55%. With Avicel as the substrate, ethanol titers increased from 1.57 to 3.24 g/l, representing an enhancement of 106%.

## Teaser

A yeast consortium system was developed to secrete and assemble five types of cellulases on yeast cell surface for improving cellulosic ethanol production.

## Introduction

Lignocellulose is the largest biomass resource on Earth and has a great potential for transformation into green biofuels and value-added chemicals (Reshmy et al. 2022). It plays a crucial role in driving the development of the biorefining industry and sustaining the global carbon cycle (Kerr 2008, Wareing et al. 2021, Reshmy et al. 2022). Consolidated bioprocessing (CBP) is a low-cost strategy for the bioproduction of second-generation biofuels (i.e. ethanol) that combines the production of cellulases, saccharification of cellulose, and fermentation into a single step (Fan et al. 2016). As a traditional ethanol-producing microorganism, *Saccharomyces cerevisiae* has been widely considered an ideal host for CBP (Sharma et al. 2022). However, the practical application of cellulosic ethanol is challenging due to the high cost of cellulases required for cellulose hydrolysis and the recalcitrant nature of cellulosic materials (Himmel 2007, Lynd et al. 2008, Raj et al. 2022).

The known essential enzymes for degrading crystalline cellulose include cellobiohydrolase (CBH), endoglucanase (EG), and *β*-glucosidase (BGL) (Wen et al. 2010). However, traditional enzymatic degradation relying on the synergistic action of CBH, EG, and BGL exhibits low efficiency in the crystalline regions of cellulose substrates; thus, pretreatment of polysaccharide substrates is required to improve the degradation efficiency of glycoside hydrolases (Lambertz et al. 2014). In 2010, Vaaje-Kolstad et al. (2010) found that lytic polysaccharide monooxygenases (LPMOs) catalyse the oxidative cleavage of cellulose in the presence of oxygen and reducing agents. This discovery opened a new avenue for enzymatic degradation of lignocellulose. Furthermore, recent studies have shown that LPMO is a cellulose-degrading enzyme with great potential, as it destroys the crystalline structure of cellulose via oxidative cleavage, assisted by enzymatic electron donors, such as cellobiose dehydrogenases (CDHs). It plays a vital role in efficient enzymatic hydrolysis of cellulose (Villares et al. 2017, Singhania et al. 2021) and has been named a “cellulase booster.”

The construction of cellulosic yeast generally follows three main strategies: secretion, cell surface display, and the mini-cellulosome system (Liu et al. 2016). Secreted cellulases can reach the secondary cell wall of the biomass to enhance the accessibility of cellulose; however, dispersed enzymes cannot be recycled (Liu et al. 2015). The cell surface maximizes synergistic interactions among different cellulases and enables enzyme recycling. However, compared with secreted enzymes, this immobilized system may affect cellulase activity, thus decreasing the degradation efficiency of cellulose (Bae et al. 2015). In contrast, the mini-cellulosome system features a highly ordered architecture that promotes proximity synergy between cellulases and cellulase–substrate–microbe complex synergy, demonstrating a significantly greater potential for industrial applications (Goyal et al. 2011, Wen et al. 2010) successfully developed the first functional mini-cellulosome on the yeast cell surface and achieved simultaneous saccharification and fermentation of cellulose to bioethanol (Wen et al. 2010). However, two critical bottlenecks hinder the further application of such cellulosomes. First, current cellulosome designs cannot achieve efficient cellulase expression. Second, the growing number of enzymes identified for biomass degradation imposes an excessive metabolic burden when expressed in a single host strain. These practical hindrances call for the development of novel expression strategies to construct superior cellulosic yeast strains for the production of second-generation fuel ethanol.

Microbial consortia are a novel strategy that leverage systematic synergy to achieve biological functions through engineered microbial communities. This approach alleviates the metabolic burden in single-strain systems by distributing biosynthetic tasks across specialized strains, and has emerged as a research frontier in recent years (Duncker et al. 2021, Wu et al. 2021) designed *Escherichia coli*–*S. cerevisiae* consortia for strigolactone biosynthesis by engineering and distributing different pathways into different strains (Wu et al. 2021). Liu et al. (2022) developed *E. coli* –*S. cerevisiae* consortia to establish a biosynthetic platform for *de novo* production of hydroxytyrosol. Honjo et al. (2019) constructed a microbial consortium system for isopropanol production from cellobiose containing different strains to sequentially perform each step of the metabolic pathway. Synthetic microbial consortium systems play a key role in enabling a variety of microorganisms to coordinate their overall function effectively (Faust 2019). Through ingenious design, researchers have enhanced the yield of metabolic pathways (Dinh et al. 2020) and achieved the cooperative production of a variety of protein products (Villarreal et al. 2018).

In this study, we developed an engineered yeast consortium system to assemble pentafunctional mini-cellulosomes on cell surfaces and improve cellulosic ethanol production based on the highest synergistic enzymatic activities. This system involves one display strain to provide the scaffoldin on the surface, and several secretion strains to secrete each cellulase. Secreted dockerin-containing enzymes, including CBH, EG, BGL, CDH, and LPMO, spontaneously assembled on the scaffoldin via specific cohesin–dockerin interactions, forming the functional pentafunctional mini-cellulosome. The novel consortium system exhibited superior characteristics compared with the conventional system (a single yeast strain secreting and displaying all the components of the five-enzyme mini-cellulosome). This study provides new ideas for designing a novel cellulosome system that may provide an economically viable solution for overcoming biomass-to-biofuel conversion bottlenecks.

## Materials and methods

### Strains, media, and reagents

The main strains and plasmids used in this study are summarized in Table 1. *Escherichia coli* DH5α was used for recombinant vector amplification in LB + ampicillin (100 μg/ml) medium. *Saccharomyces cerevisiae* SK1, constructed in our previous study (Song et al. 2017), was used as the starting strain for the construction of the engineered yeast strains. The SK-CBHI strain was constructed by transforming the CBHI expression cassette from pBS-CBHI using the δ-integration strategy, as were the other strains. The SK-control strain was constructed by transforming five different expression cassettes, and the expression of each enzyme component in the five-enzyme mini-cellulosome was confirmed by enzyme activity verification or polymerase chain reaction (PCR) amplification. The constructed yeast strains were selected and grown on yeast extract peptone dextrose (YPD) medium containing yeast extract (10 g/l), peptone (20 g/l), and glucose (20 g/l). DNA extraction and purification kits were purchased from Zymo Research (Irvine, CA). Restriction enzymes and DNA polymerase were purchased from New England Biolabs (Ipswich, MA). The PCR primers were synthesized by Integrated DNA Technologies (IDT; Coralville, IA). All chemicals were purchased from Fisher Scientific (Pittsburgh, PA) and Sigma-Aldrich (St. Louis, MO).

**Table 1.** Characteristics of yeast strains and main plasmids used in this study.

Plasmids and strains	Relevant features	Origin
Strains		
SK1	*MATa his3*Δ1 *leu2*Δ0 *met15*Δ0 *ura3*Δ0 *tpi1::loxP*	Song et al. (2017)
SK6(Control -)	δ-integration of pBS12, as the control strain	Song et al. (2018)
SK-control	A single yeast strain, which displays each component enzyme of the five-enzyme mini-cellulosome.	In this study
SK-CBHI	δ-integration of expression cassette from pBS-CBHI	In this study
SK-EGII	δ-integration of expression cassette from pBS-EGII	In this study
SK-BGLI	δ-integration of expression cassette from pBS-BGLI	In this study
SK-CDH	δ-integration of expression cassette from pBS-CDH	In this study
SK-LPMO	δ-integration of expression cassette from pBS-LPMO	In this study
SK-CipA5-AGA1	δ-integration of expression cassette from pBS-CipA5-AGA1	In this study
Plasmids		
pBS12	pBluescript II SK (+)-δ1*-POT1*-δ2	Song et al. (2018)
pBS-CBHI	pBluescript II SK (+)-δ1*-TPI1p*-*CBHI*-*Dockerin*-*TPI1t*-*POT1*-δ2	In this study
pBS-EGII	pBluescript II SK (+)-δ1*-TPI1p*-*EGII*-*Dockerin*-*TPI1t*-*POT1*-δ2	In this study
pBS-BGLI	pBluescript II SK (+)-δ1*-TPI1p*-*BGLI*-*Dockerin*-*TPI1t*-*POT1*-δ2	In this study
pBS-CDH	pBluescript II SK (+)-δ1*-TPI1p*-*CDH*-*Dockerin*-*TPI1t*-*POT1*-δ2	In this study
pBS-LPMO	pBluescript II SK (+)-δ1*-TPI1p*-*LPMO*-*Dockerin*-*TPI1t*-*POT1*-δ2	In this study
pBS-CipA5-AGA1	pBluescript II SK (+)-δ1*-TPI1p*-*CipA5-AGA1*-*Dockerin*-*TPI1t*-*POT1*-δ2	In this study
pBS18	pUC19 with *ALG9, EGII* genes used for qPCR standard curve analysis	Song et al. (2018)
pBS19	pUC19 with *ALG9, BGLI* genes used for qPCR standard curve analysis	Song et al. (2018)
pBS20	pUC19 with *ALG9, CBHI* genes used for qPCR standard curve analysis	Song et al. (2018)
pBS21	pUC19 with *ALG9, CDH* genes used for qPCR standard curve analysis	In this study
pBS22	pUC19 with *ALG9, LPMO* genes used for qPCR standard curve analysis	In this study
pBS23	pUC19 with *ALG9, CipA5* genes used for qPCR standard curve analysis	In this study

### Plasmid construction

The PCR primers used are summarized in Table S1 and the genetic characteristics of the cellulase-expressing plasmids are shown in Fig. S1. PCR products containing the signal peptides Yap3-TA57 (amplified from pBS17 with primer pair ss-F/R), *Talaromyces emersonii* CBHI gene (amplified from pBS17 with primer pair tCBH-F/R), and DocS (the dockerin domain of *Clostridium thermocellum* CelS amplified from pRS426-CBHI with primer pair docS-F/R) were obtained through fusion PCR and ligated into *Xba*I/*Sac*II digested pBS17 to generate pBS-CBHI (Fig. S1). pBS17 and pRS426-CBHI were constructed in a previous study (Song et al. 2018). pBS-CipA5-AGA1 was constructed using a process similar to that described above. The CipA5-AGA1 fused gene was amplified from pRS426-CipA5 and ligated into *Xba* I/*Sac* II-digested pBS17 to generate pBS-CipA5-AGA1. Other cellulase genes from *Trichoderma reesei* EGII, *Aspergillus aculeatus* BGLI, *Thermoascus aurantiacus* GH61a (LPMO), and *Humicola insolens* CDH were obtained through PCR from pBS15, pBS16, pRS426-LPMO, and pRS426-CDH, respectively, and individually ligated into *Sbf* I/*Not* I*-*digested pBS-CBHI to generate pBS-EGII, pBS-BGLI, pBS-LPMO, and pBS-CDH, respectively. Note that the epitope tag was fused to the N-terminus of each cellulase gene, which allowed surface display and assembly efficiency detection through flow cytometry analysis. Cellulase gene expression cassettes were transformed and integrated into the chromosome of *S. cerevisiae* SK1 using a previously described chemical transformation method (Gietz and Woods 2002).

### Function analysis of cellulases in the pentafunctional mini-cellulosome

To ensure the successful implementation of the yeast consortium system, the cellulose degradation capacities of the control system (SK6 supernatant), three-enzyme system (a supernatant mix from SK-CBHI, SK-EGII, and SK-BGLI), and five-enzyme system (a supernatant mix from SK-CBHI, SK-EGII, SK-BGLI, SK-CDH, and SK-LPMO) in hydrolysis media containing 1% phosphoric acid swollen cellulose (PASC) were compared. The yeasts were cultivated for 72 h at 30°C in 50 ml YPD medium. Then, crude enzymes (supernatants) collected through centrifugation were used for cellulose degradation. After 6 h of cultivation in PASC-containing medium, the residual amount of insoluble PASC was collected through centrifugation for direct observation. To quantify the hydrolytic activity of the different systems, the glucose assay kit (Solarbio^®^ BC2500, Beijing) was used for analysis of the amount of residual glucose.

### Yeast surface display and flow cytometry analysis

To verify the anchorage of the miniscaffoldin (CipA5) on the yeast cell surface of the SK-CipA5-AGA1 strain, the cells were labeled with a FLAG antibody, followed by flow cytometry analysis as described previously (Wen et al. 2008). To verify the scaffoldin assembly ability of other cellulases in the pentafunctional mini-cellulosome, the supernatants of the control strain SK6 and five cellulase-secreting strains were mixed with SK-CipA5-AGA1. The mixed cells were then labeled with a single type of antibody for each flow cytometry analysis. The docking of CBHI, EGII, BGLI, CDH, and LPMO onto the surface-displayed miniscaffoldin CipA5 was verified by detecting Myc, 6 × His, HA, T7, and V5 tag epitopes, respectively. Approximately 2.5 × 10^6^ cells were used for flow cytometry analysis. The appropriate fluorescence conjugation kits and primary monoclonal antibodies were purchased from Invitrogen.

### Quantitative PCR

The copy numbers of cellulase genes were analysed by comparing *Ct* values of the target and reference genes using a previously reported method (Shi et al. 2014). As shown in Table 1, the plasmid pBS18 was used as the template for the analysis of the EGII/ALG9 quantitative PCR (qPCR) standard curve, while pBS19 was used for BGLI, pBS20 for CBHI, pBS21 for CDH, pBS22 for LPMO, and pBS23 for CipA5. The target genes were amplified using primer pairs QEG2-F/R, QBGL1-F/R, QCBH1-F/R, QCDH-F/R, QLPMO-F/R, and QCipA5-F/R, respectively. The reference gene ALG9 was amplified using the primer pair QALG9-F/R (Table S1). The qRT-PCR program was set as follows: 95°C for 30 s, followed by 40 cycles of 5 s at 95°C, 30 s at 57°C, and 30 s at 72°C. A CFX Connect Real-Time PCR Detection System (Bio-Rad, Hercules, CA) was used for melting curve and qPCR analyses.

### Enzyme activity assay

Yeast cells were cultivated for 72 h at 30°C in 50 ml YPD medium. Then, crude enzymes (supernatants) collected by centrifugation were used for enzyme activity analysis. CBHI, EGII, and BGLI activities were determined using a previously reported method (Liu et al. 2015). One unit of CBHI/BGLI activity was defined as the enzyme amount required for production of 1 μM *p*-nitrophenol (*p*NP) at 50°C/min. One unit of EGII activity was defined as the absorption at 590 nm of released blue dye at 38°C/min.

### Fermentation

The cells with an OD_600_ of 50 were cultured in YP-PASC/Avicel medium containing yeast extract (10 g/l), peptone (20 g/l), and PASC/Avicel (1%). As the function of LPMO requires oxygen, fermentation was performed under microaerobic conditions, and the cells were cultivated in a 30 ml serum bottle with 5% volumetric air headspace at 37°C with agitation at 250 rpm. High-performance liquid chromatography (HPLC) (Finnigan Surveyor Plus, Thermo Scientific) and a refractive index (RI) detector (Finnigan Surveyor RIPlus detector, Thermo Scientific) were used to quantify the ethanol concentration. The BioRad Aminex HPX-87H column (300 mm × 7.8 mm, 5 μm) was applied for HPLC analysis and the program was set as follows: 5 mM H_2_SO_4_ as eluent, 60°C, with a flow rate of 0.8 ml/min.

## Results

### Design and construction of an engineered yeast consortium displaying a pentafunctional mini-cellulosome

**Figure 1.**
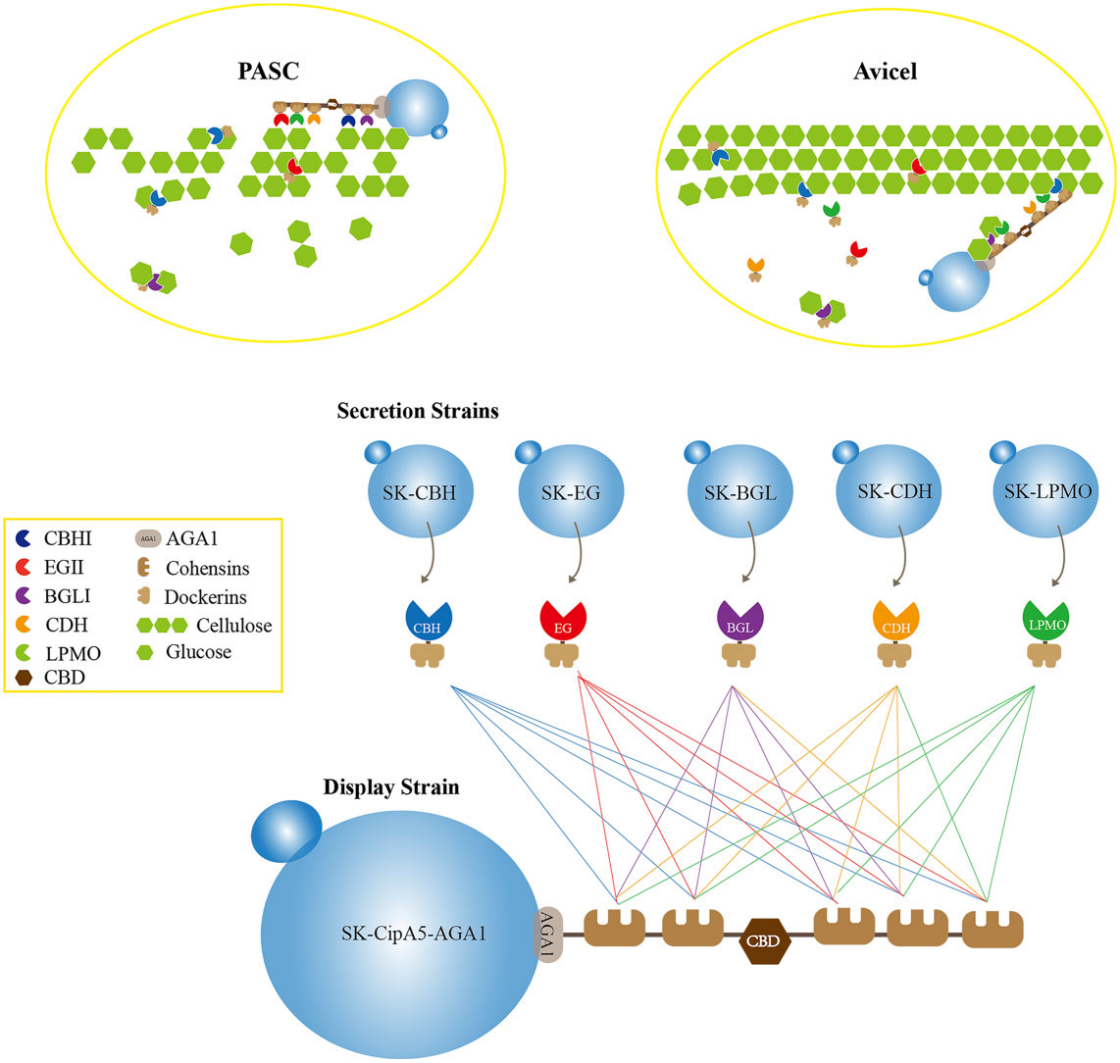
Schematic representation of an engineered yeast consortium displaying a pentafunctional mini-cellulosome.

The synergistic action of various cellulases greatly improves degradation efficiency; however, when overloaded cellulase genes are expressed in cells, the metabolic burden on the cells may greatly affect cell growth and cellulosic ethanol production (Song et al. 2018). In this study, we developed a yeast consortium system that displayed a pentafunctional mini-cellulosome (Fig. 1). The novel consortium system contained six different yeast strains, one for displaying the miniscaffoldin CipA5 and the other five strains for secreted expression of each type of cellulase (CBH, EG, BGL, CDH, and LPMO). CipA5 is a modified miniscaffoldin cloned from *C. thermocellum* that contains a CBM and five type I cohesin domains (Olson et al. 2013). In this study, CipA5 was expressed on the surface of the yeast SK1 strain by fusing with the Aga1-fusion protein. To obtain high-performance strains secreting different cellulases, different expression cassettes were designed, each containing a promoter, secretion signal peptide, epitope tag, cellulase, dockerin module, and transcription terminator. The cellulases were then docked onto the surface-displayed miniscaffoldin using dockerin modules. Subsequently, the secreted dockerin-containing cellulases were spontaneously assembled to the scaffoldin to generate the pentafunctional mini-cellulosome via the interactions of cohesin and dockerin.

### Functional analysis of cellulases in the pentafunctional mini-cellulosome

**Figure 2.**
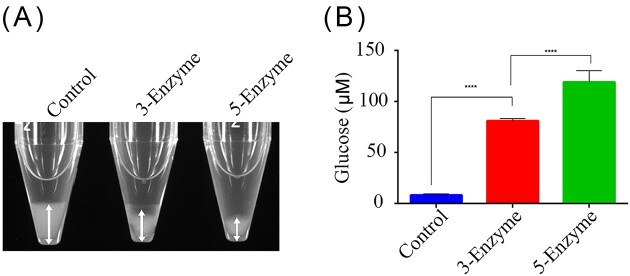
Function analysis of the cellulases. (A) Cellulose degradation capacity analysis. The different system were cultured in YP-PASC medium (1% PASC) for 6 h at 50°C. (B) The analysis of glucose production. The five-enzyme system had significantly higher cellulose hydrolysis activity, as indicated by the larger amounts of glucose released. Asterisks indicate significant differences from the control strain (*P < .05; **P < .01; ***P < .001; ^****^P < .0001, Student's *t* test).

The functions of three essential cellulases were systematically characterized in our previous study (Song et al. 2018). To ensure successful implementation of the yeast consortium system, the cellulose degradation capacities of the control system (SK6 supernatant), three-enzyme system (a supernatant mix of SK-CBHI, SK-EGII, and SK-BGLI), and five-enzyme system (a supernatant mix of SK-CBHI, SK-EGII, SK-BGLI, SK-CDH, and SK-LPMO) in hydrolysis medium containing 1% PASC were compared. As shown in Fig. 2(A), after 6 h of cultivation in PASC-containing medium, both the three- and five-enzyme systems hydrolysed cellulose. Compared with the three-enzyme system, the residual amount of PASC in the five-enzyme system was lower, indicating that PASC was hydrolyzed to a greater extent by the five-enzyme system expressing LPMO and CDH. These results illustrated that LPMO and CDH facilitated the hydrolysis of cellulose. Then, the hydrolytic activity was further quantified by measuring the amount of residual glucose. As shown in Fig. 2(B), the glucose levels generated by PASC hydrolysis differed significantly between the two systems. The five-enzyme system generated a glucose content of 118.76 μM, which was higher than the 80.88 μM generated by the three-enzyme system, representing an enhancement of 47% in the cellulose hydrolysis activity.

### Assembly efficiency analysis of the cellulases in pentafunctional mini-cellulosome

**Figure 3.**
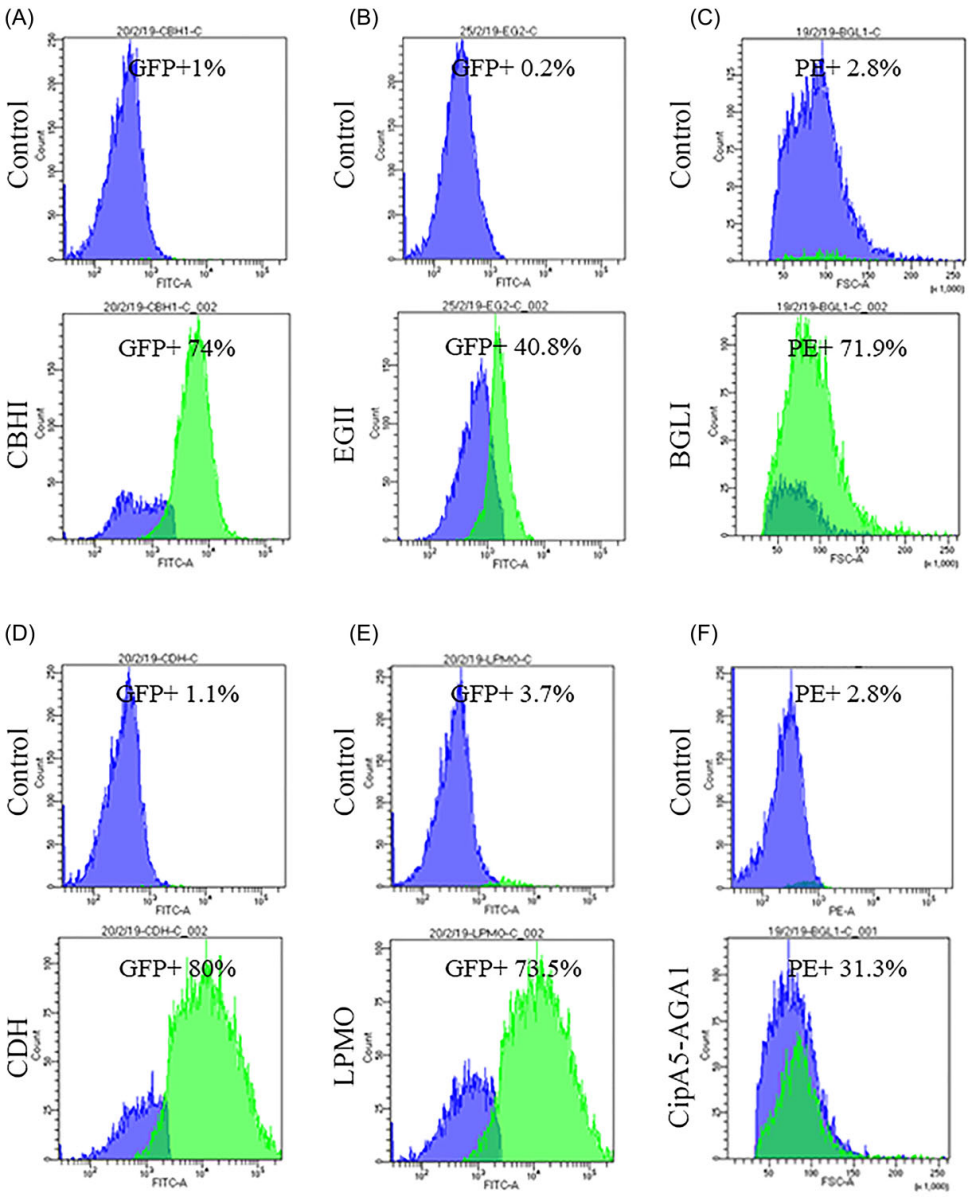
Flow cytometry analysis of mini-cellulosome-assembly-capacity of cellulases. (A–E) Flow cytometry analysis of mini-cellulosome-assembly-capacity of CBHI, EGII, BGLI, LPMO and CDH. The enzymes were expressed, secreted, and docked onto the miniscaffoldins, as indicated by the Myc, 6 × His, HA, T7 and V5 detection. (F) Flow cytometry analysis of CipA5-AGA1 miniscaffoldins. CipA5-AGA1 were successfully displayed on the yeast cell surface, as indicated by Flag epitope detection. The control strains used in this figure were all SK6.

The assembly capacity of each cellulase was tested to ensure successful implementation of the pentafunctional mini-cellulosome. To verify the anchorage of the miniscaffoldin (CipA5) on the cell surface in the SK-CipA5-AGA1 strain, cells were labeled with an anti-FLAG antibody, followed by flow cytometry analysis. As indicated by FLAG epitope detection, the miniscaffoldin CipA5 was successfully expressed on the SK-CipA5-AGA1 cell surface (Fig. 3F). To verify the scaffoldin assembly ability of cellulases in the pentafunctional mini-cellulosome, the supernatants of the control strain SK6 and five cellulase-secreting strains were mixed with SK-CipA5-AGA1. SK-CipA5-AGA1 cells were then labeled with the corresponding antibody for each flow cytometry analysis. The docking of CBHI, EGII, BGLI, CDH, and LPMO onto the surface-displayed miniscaffoldin CipA5 was verified by detecting Myc, 6 × His, HA, T7, and V5 tag epitope, respectively. Flow cytometry results showed that all SK-CipA5-AGA1 cells mixed with the supernatants of the five cellulase strains were antigen-positive, which systematically validated the assembly ability of the pentafunctional mini-cellulosome (Fig. 3).

### Screening superior cellulase-expressing strains used for yeast consortium system

**Figure 4.**
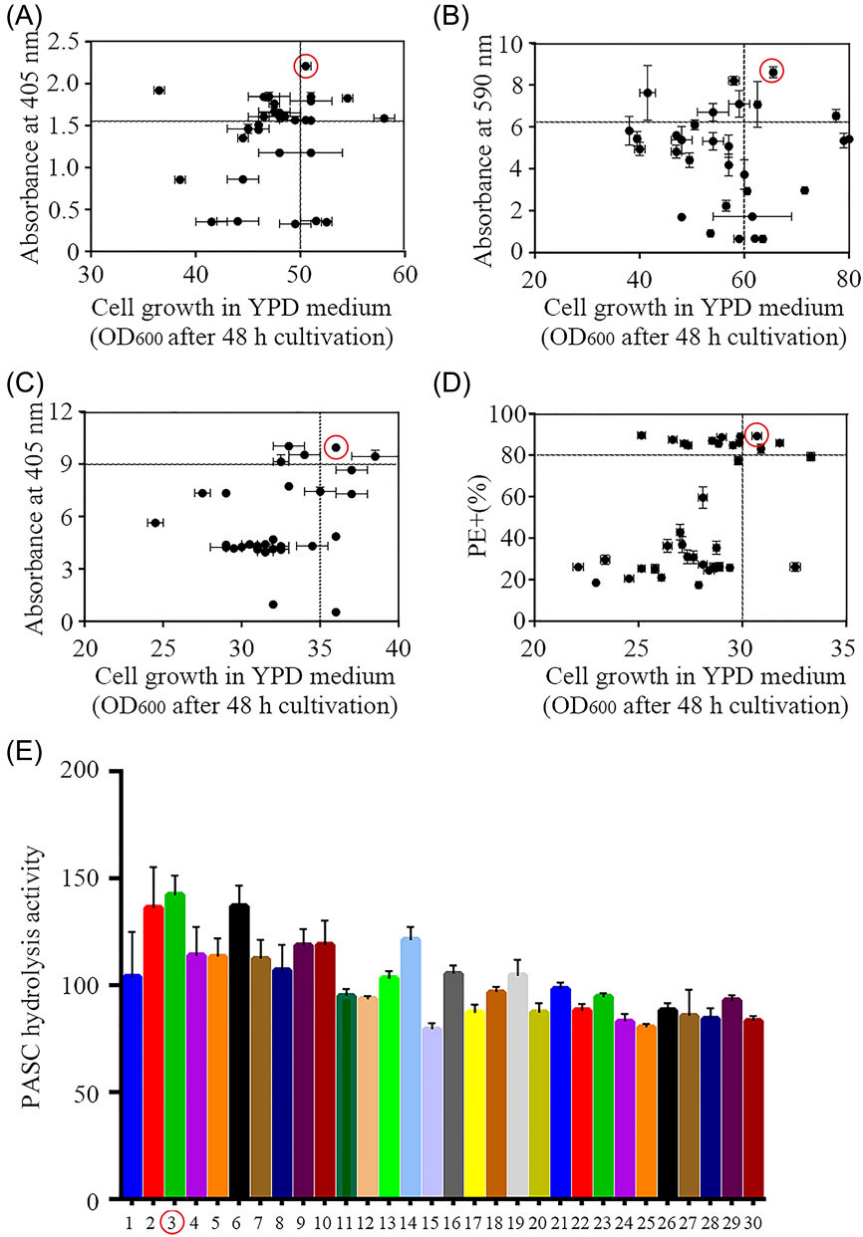
Screening of highly expressed strains for yeast consortium system. (A) Cell growth and enzyme activity of CBHI-expressing strains. (B) Cell growth and enzyme activity of EGII-expressing strains. (C) Cell growth and enzyme activity of BGLI-expressing strains. (D) Cell growth and PE staining of CipA5-expressing strains. (E) PASC hydrolysis of CDH and LPMO combination-strains. The strains circled in red are the highly expressed strains screened for yeast consortium system.

The cellulase genes were heterologously expressed using the *POT1*-mediated *δ*-integration method (Song et al. 2017). Integration of cellulases simultaneously occurs on multiple chromosomes due to the presence of about 425 *δ*-regions dispersed in the genome of *S. cerevisiae*, which provide the opportunity for integrating multiple copies of cellulase genes into the yeast genome (Yamada et al. 2010). The expression level of cellulase directly affects the cellulose degradation efficiency. However, excessive protein expression in cells generally decreases cell growth and respiratory capacity, and such growth-deficient strains are generally unsuitable for industrial application (Liu et al. 2017). Thus, for each high-performance cellulase-expressing strain, 30 transformants were selected as candidates, and two factors—growth rate and enzyme activity—were considered during the selection process. Considering the synergistic function of LPMO and CDH, their screening was performed using PASC hydrolysis activity in combination. As shown in Fig. 4, the strains circled in red represent high-performance cellulase-expressing strains selected for the yeast consortium system.

### Advantage evaluation of the novel consortium system

**Figure 5.**
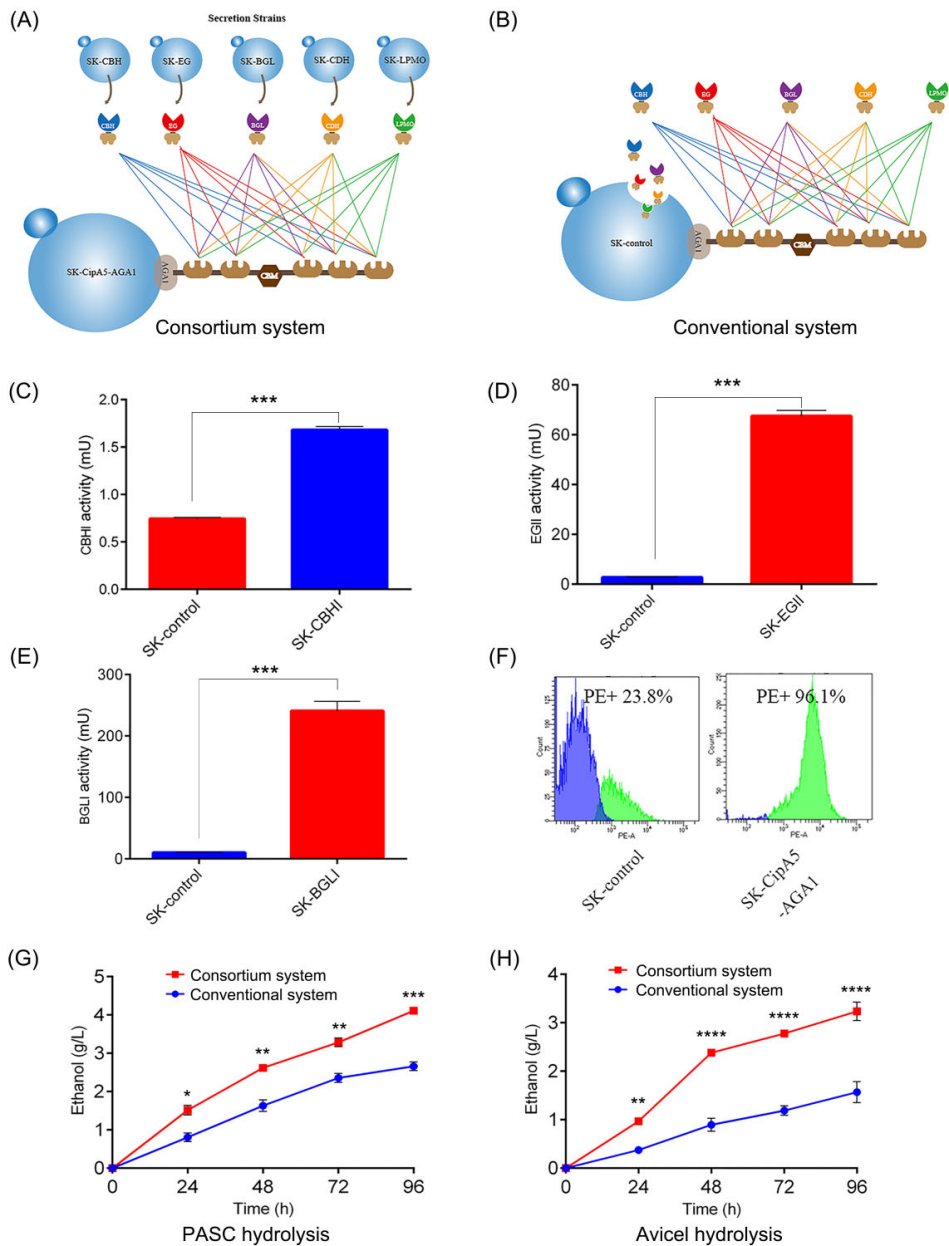
Comparison of two different systems. (A) Consortium system: an engineered yeast consortium displaying a pentafunctional mini-cellulosome. (B) Conventional system: a single yeast strain (SK-control), which secretes and displays each component enzyme of the five-enzyme mini-cellulosome. (C) CBHI activity. (D) EGII activity. (E) BGLI activity. (F) Assembly efficiency. (G) Ethanol production with PASC as the substrate. (H) Ethanol production with Avicel hydrolysis. Asterisks indicate significant differences from the control strain (*P < .05; **P < .01; ***P < .001; ^****^P < .0001, Student's *t* test).

To further demonstrate the superiority of the consortium system, strain growth, copy number of cellulase genes, enzyme activity, assembly efficiency, and ethanol production were tested in both the conventional and consortium systems. A schematic representation of the consortium system is shown in Fig. 5(A), and the conventional system or a single yeast strain (SK-control) secreting and displaying all the components of the five-enzyme mini-cellulosome is shown in Fig. 5(B). The consortium was composed of an equal ratio of each component.

When overloaded cellulase genes are expressed in cells, the metabolic burden on the cells may greatly affect cell growth and cellulosic ethanol production (Song et al. 2018). Therefore, the physiological characteristics of the cellulolytic yeast strains in the two systems were initially investigated (Fig. S2). In addition, the enzyme activities of three essential cellulases and the assembly efficiency of the scaffoldin were compared in two different systems (Fig. 5C–F), using the same biomass. As shown in Fig. 5(C), the CBHI activity of the screened CBHI-expressing strain SK-CBHI in the consortium system was 1.68 mU, yielding a 2.27-fold increase compared to 0.74 mU from the SK-control strain in the conventional system. The EGII activity of SK-EGII in the consortium system was 67.4 mU, displaying a 24-fold increase compared to 2.8 mU from the SK-control strain in the conventional system (Fig. 5D). The BGLI activity of the SK-BGLI strain was 241 mU, which was a 23-fold increase compared to 10.5 mUfrom the SK-control (Fig. 5E). The assembly efficiency achieved with the screened CipA5-expressing strain SK-CipA5-AGA1 was significantly higher than that of the SK-control strain (Fig. 5F). These significant increases highlighted the superiority of the constructed consortium system, which was probably due to the relief of the metabolic burden placed on the engineered single yeast strain.

The consortium system displaying a pentafunctional mini-cellulosome was further compared to the conventional system to evaluate its ethanol production capacity directly from cellulose. In this study, two types of cellulosic substrates were used, PASC (as a pretreated cellulose) and Avicel (as a microcrystalline cellulose), which are widely used to produce cellulosic ethanol. The results showed that the maximum ethanol titre achieved was 4.11 g/l in the constructed consortium system and 2.66 g/l in the conventional system for PASC degradation (Fig. 5G), an improvement of 55%. When Avicel was used as the substrate, the maximum ethanol titre was 3.24 g/l in the constructed novel consortium system and 1.57 g/l the in conventional system (Fig. 5H), an improvement of 106%. It is likely that the enzymes in the consortium system acted synergistically and efficiently to boost cellulose degradation and ethanol production.

## Discussion

Sustainable utilization of cellulosic biomass to produce value-added products is an ideal strategy, but hydrolysis of recalcitrant cellulose is time-consuming and complex (Liu et al. 2019, Qiao et al. 2022). As a multienzyme machinery, cellulosomes are considered the best cellulose degradation machinery in nature (Lamote et al. 2023). In recent years, several studies have successfully constructed cellulosomes from *S. cerevisiae* and demonstrated their ability to hydrolyse cellulose for ethanol production (Wen et al. 2010, Fan et al. 2012, 2016, Tsai et al. 2013). The synergistic action of various cellulases greatly improves the degradation efficiency; however, when overloaded cellulase genes are expressed in one cell, the metabolic burden on the cell may greatly affect cell growth and cellulosic ethanol production (Song et al. 2018). Moreover, the highly efficient and diverse expression of cellulase in cellulosomes is extremely challenging owing to the limitations of cellulosome design in one host cell.

The consortium system, which flexibly modularizes and divides an entire path among member strains to balance the biosynthesis ability between modules, is a rising modality in microbial biosynthesis research (Morais et al. 2014, Mai et al. 2023). In this study, we developed a consortium system displaying a pentafunctional mini-cellulosome to improve cellulosic ethanol production. As shown in Fig. 5, the developed system decreased the metabolic burden of the yeast host by distributing the metabolic burden to multiple strains, but preserved the ability of spontaneous assembly and display of mini-cellulosomes. In terms of ethanol production, Fan et al. (2012) achieved 1.41 g/l and 1.09 g/l of ethanol from Avicel and PASC, respectively, using the engineered mini-cellulosome system. Liang et al. (2014) enhanced ethanol production to 1.5 g/l and 2.7 g/l for Avicel and PASC, respectively, by introducing CDH and LPMO. In this study, we attained higher ethanol titres, 3.24 g/l with Avicel as the substrate and 4.11 g/l for PASC hydrolysis. In addition, existing approaches generally express mini-cellulosomes for higher protein yields using episomal plasmids, but face stability concerns. Here, the cellulosome genes were integrated onto the yeast genome using the *POT1*-mediated δ-integration strategy (Song et al. 2017). In the *POT1* system, the conventional rich complex medium enforced the stable expression of high-copy cellulase genes, increasing cell numbers and protein production, which is beneficial for industrial applications.

Despite the promising results obtained in this study, several improvements can be made to engineer these strains in the future. Cellulosome activity and ethanol production in the constructed consortium system could be improved by optimizing the combination ratio of the six yeast strains via a mixture experimental design. Accordingly, the synergistic mechanisms of cellulases should be explored to significantly enhance our understanding of the engineering of CBP systems for the production of cellulosic ethanol from biomass. In addition, the consortium system alleviates the metabolic load on a single host by dispersing labor over several enzymes, providing a strategy for the application of other types of enzymes (i.e. hemicellulases and laccases) into the cellulosome complex. Furthermore, any enzyme gene can be fine-tuned using an enzyme engineering strategy to realize the overall optimization of the consortium system by leveraging the modularity of the developed system.

In conclusion, we engineered a yeast consortium that secretes and assembles five cellulases on the yeast surface to form pentafunctional mini-cellulosome for cellulosic ethanol production. Cellulases in pentafunctional mini-cellulosomes exhibit highly ordered structural organization and assembly, which enables cellulase proximity-driven synergy and cellulase–substrate–microbe complex synergy, showing much greater industrial application potential. We envision that this novel consortium design can be applied to the biosynthesis of industrial products containing multienzyme cascade biomanufacturing, which will demonstrate the transformative potential in the application of yeast as a cell factory for the bioproduction of heterologous proteins of medical or industrial interest.

## Supplementary Material

foaf022_Supplemental_File Additional file: Fig. S1. Genetic properties of cellulase expressing plasmids. Figure S2. The physiological characteristics of the cellulolytic yeast strains in two different systems. (A) Cell growth of cellulolytic yeast strains. (B) The copy numbers of the genes expressed by strains in conventional system and the novel consortium system. Table S1. Polymerase chain reaction primers used in this study.

## Data Availability

All data and materials supporting the findings of this study are included in the article and its additional files.
