# Supplementary material for: Improving cellulosic ethanol production by an engineered yeast consortium displaying a pentafunctional mini-cellulosome
Source: FEMS Yeast Res. 2025 May 21;25:foaf022. doi: 10.1093/femsyr/foaf022 (PMC12108760; doi:10.1093/femsyr/foaf022)
Supplement: foaf022_Supplemental_File — Additional file: Fig. S1. Genetic properties of cellulase expressing plasmids. Figure S2. The physiological characteristics of the cellulolytic yeast strains in two different systems. (A) Cell growth of cellulolytic yeast strains. (B) The copy numbers of the genes expressed by strains in conventional system and the novel consortium system. Table S1. Polymerase chain reaction primers used in this study. [file foaf022_supplemental_file.docx]

**ADDITIONAL FILE**

**Improving Cellulosic Ethanol Production by an Engineered Yeast Consortium Displaying a Pentafunctional Mini-cellulosome**

Xiaofei Song^1^, Jianze Zhang^1^, Siyu Fu^1^, Ziyi Liu^1^, Yan Chen^1^, Tingheng Zhu^1*^

^1^ College of Biotechnology and Bioengineering, Zhejiang University of Technology, Hangzhou 310014, Zhejiang Province, China


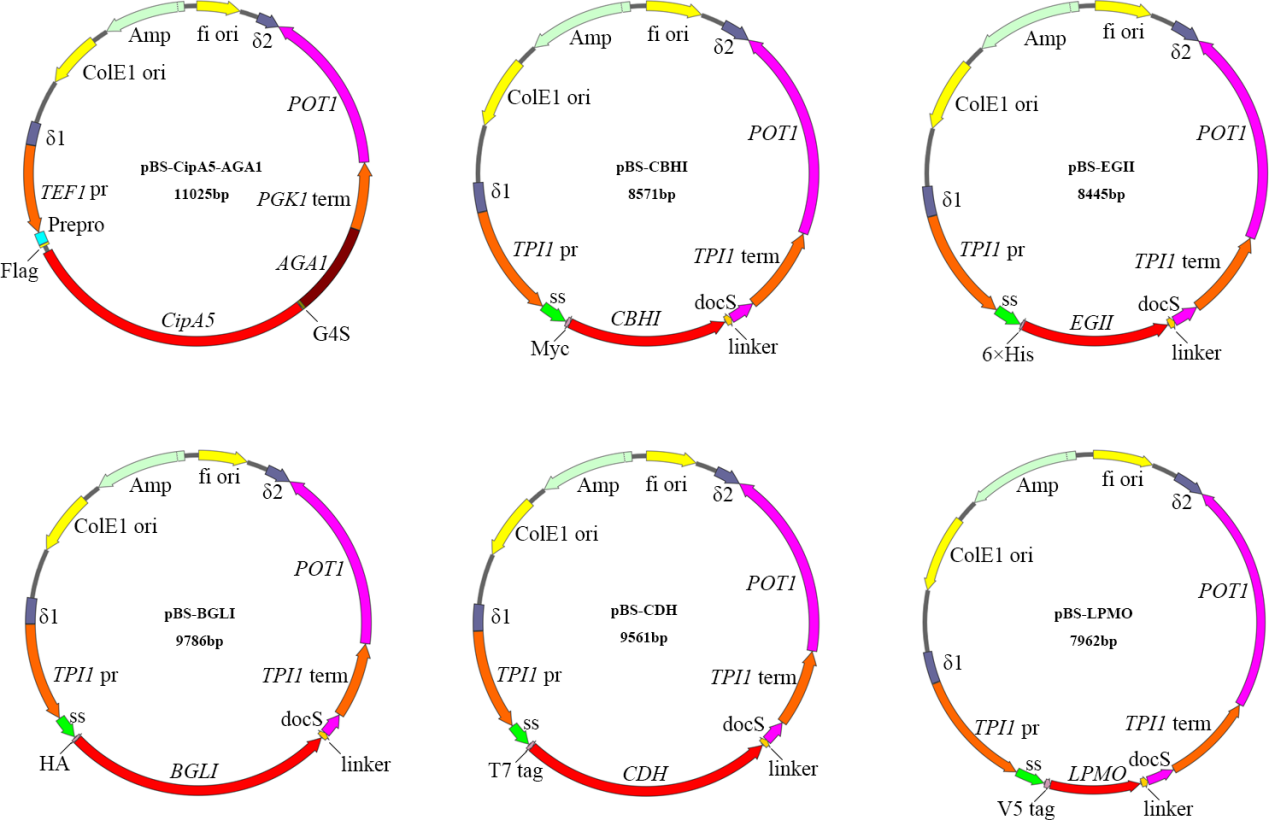


**Fig. S1.** Genetic properties of cellulase expressing plasmids.


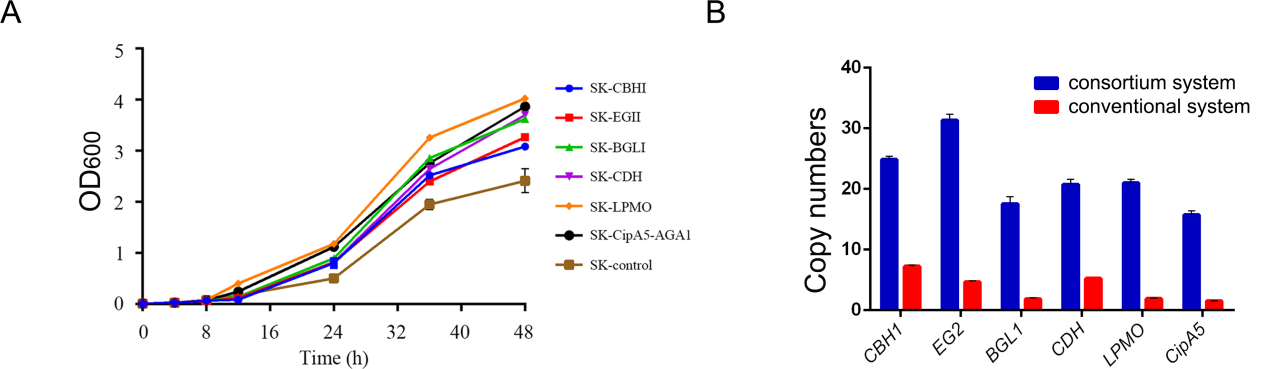


**Fig. S2.** The physiological characteristics of the cellulolytic yeast strains in two different systems. (A) Cell growth of cellulolytic yeast strains. (B) The copy numbers of the genes expressed by strains in conventional system and the novel consortium system.

**Table S1** Polymerase chain reaction primers used in this study.

| **Primers** | **Sequence (5’- 3’)** | **Source of DNA sequence** |
| --- | --- | --- |
| **docS-F** | TCTTAGTTTCTAGACTGCAGTTAGTTCTTGTACGGCAATG | pRS426-CBH1 |
| **docS-R** | TGGTGGTGGTGGTTCTTCTACTAAATTATACGGCGAC |  |
| **tCBH-F** | ATTTAGTAGAAGAACCACCACCACCAGAACCACCACCACCAGAACCACCACCACCACTAGCACCTGCAGGAGAAGCAGTGAAAGTGGAGTTGATTG | pBS17 |
| **tCBH-R** | TCTCAGAAGAGGATCTGGCCGGTACCGCTACTGCT |  |
| **ss-F** | CGGTACCGGCCAGATCCTCTTCTGAGATGAGTTTTTGTTCGCGGCCGCCTTTTGGTTCACCTTCTTCTCTTTTAG | pBS17 |
| **ss-R** | TCCCCGCGGAACAAAATGAAATTGAAAACTGT |  |
| **tEG-F** | ACTAGCACCTGCAGGCTTTCTAGCTAAACACGATGAGAC | pBS15 |
| **tEG-R** | AAGGAAAAAAGCGGCCGCCATCATCACCATCACCATCAACAAACCGTATGGGGTC |  |
| **tBGL1-F** | ACTAGCACCTGCAGGCTGAACCTTAGGCAAAGCAG | pBS16 |
| **tBGL1-R** | AAGGAAAAAAGCGGCCGCTACCCATACGATGTTCCAGATTACGCTGATGAGTTGGCTTTTTCTCC |  |
| **tCDH-F** | ACTAGCACCTGCAGGCTTCCTCAGCTTAAGAATGC | pRS426-CDH |
| **tCDH-R** | AAGGAAAAAAGCGGCCGCATGGCTAGCATGACTGGTGGACAGCAAATGGGTGCCGCGCAAGCCACTGGT |  |
| **tLPMO-F** | ACTAGCACCTGCAGGACCAGTATACAGAGGAGGACCAGGGATGATATAGCTGGAAAGTTTCTG | pRS426-LPMO |
| **tLPMO-R** | AAGGAAAAAAGCGGCCGCGGTAAACCAATTCCAAATCCATTGTTGGGTTTGGATTCTACTGGCTTCGTTCAGAACATCGTGAT |  |
| **CipA5A-F** | CATGCCATGGAGCAACAGGCGCGTTGGAC | pRS426-CipA5 |
| **CipA5A-R** | GGAATTCCATATGCAGGAAGAATACACTATACTGGATCTA |  |
| **pALG9-F** | CCGGAATTCATGAATTGCAAGGCGGTAAC | *S.cerevisiae* genomic DNA |
| **pALG9-R** | CGCGGATCCGCCTATGATTATCTGGCAGC |  |
| **pCBH1-F** | CGCGGATCCAACAAAATGAAATTGAAAACTGT | pUC57-Yap3CBHI |
| **pCBH1-R** | AACTGCAGTTAAGAAGCAGTGAAAGTGGAG |  |
| **pEG2-F** | CGCGGATCCAACAAAATGAAATTGAAAACTGT | *­*pUC57-Yap3EGII |
| **pEG2-R** | AACTGCAGCTACTTTCTAGCTAAACACGATG |  |
| **pBGL1-F** | CGCGGATCCAACAAAATGAAATTGAAAACTGT | pUC57-Yap3BGLI |
| **pBGL1-R** | AACTGCAGTCACTGAACCTTAGGCAAAG |  |
| **pCDH-F** | CGCGGATCCCTTCCTCAGCTTAAGAATGCG | pBS-CDH |
| **pCDH-R** | AACTGCAGTGCCCCTGAGGAGACTTATG |  |
| **pLPMO-F** | CGCGGATCCATGCACTGGGGATAGTTCTG | pBS-LPMO |
| **pLPMO-R** | AACTGCAGGGCTTCGTTCAGAACATCG |  |
| **pCipA5-F** | CGCGGATCCAGCTCTTGTCAGGATTCGG | pBS-CipA5 |
| **pCipA5-R** | AACTGCAGCCGATTACTTTGCTTGAAGTAG |  |
| **QALG9-F** | GCCGTCTACGAGCAATTTTC | pBS18/pBS19/pBS20/pBS21/pBS22/pBS23/ SK-CBHI genomic DNA/ SK-EGII genomic DNA/ SK-BGLI genomic DNA/ SK-CDH genomic DNA/ SK-LPMO genomic DNA/ pBS23/ SK-CipA5 genomic DNA |
| **QALG9-R** | ATCTGGCAGCAGGAAAGAAC |  |
| **QCBH1-F** | GTGGGACGACTACGCTGCTC | pBS20/SK-CBHI genomic DNA |
| **QCBH1-R** | GGAGTTTGGAGATTGGGATTC |  |
| **QEG2-F2** | ATGCTCAATGTATTCCTGGCG | pBS18/SK-EGII genomic DNA |
| **QEG2-R2** | GTGGAGTGGAGGAAGAGGTCG |  |
| **QBGL1-F2** | AGATGGTGGTAGGAACTGGGAG | pBS19/SK-BGLI genomic DNA |
| **QBGL1-R2** | AGTGTTTAGCTGTTGCGACGAC |  |
| **QCDH-F** | GCTCTCCGTCAACATCACCG | pBS21/SK-CDH genomic DNA |
| **QCDH-R** | CACGACCGAGGACGAGATAG |  |
| **QLPMO-F** | GGCTTCGTTCAGAACATCGT | pBS22/SK-LPMO genomic DNA |
| **QLPMO-R** | TCCAAGATCAGTTGCCGTAG |  |
| **QCipA5-F** | GCAATAATCGGCAGTAACGG | pBS23/SK-CipA5 genomic DNA |
| **QCipA5-R** | TTTCAAGGTAGGTGTCTGCG |  |

Note: The restriction enzyme cutting sites are underlined.
